# Assessment of Health-Related Quality of Life of Stroke Survivors in Southeast Communities in Nigeria

**DOI:** 10.3390/ijerph21091116

**Published:** 2024-08-23

**Authors:** Gloria Ada Adigwe, Folashade Alloh, Patricia Smith, Rachel Tribe, Pramod Regmi

**Affiliations:** 1 Primary Care Physiotherapy, Beckenham PCN, Bennett Road, Leeds LS6 3HN, UK; gloria.adigwe1@nhs.net; 2 School of Health & Society, Faculty of Education, Health and Wellbeing, University of Wolverhampton, Wolverhampton WV1 1LY, UK; 3 School of Health, Sports and Bioscience, University of East London, Water Lane, London E15 4LZ, UK; p.a.smith@uel.ac.uk (P.S.); r.tribe@uel.ac.uk (R.T.); 4 Department of Nursing Sciences, Faculty of Health & Social Sciences, Bournemouth University, Fern Barrow, Poole BH12 5BB, UK; pregmi@bournemouth.ac.uk

**Keywords:** stroke, quality of life, Nigeria

## Abstract

The prevalence of stroke in Nigeria has continued to be a major public health challenge. Recovery from a stroke episode can be a long-impacting process with reduced quality of life outcomes. Past studies have explored the quality of life (QoL) of stroke survivors. However, none have explored the QoL of stroke survivors in Southeastern Nigeria. This study therefore describes the QoL of Nigerian stroke survivors in Southeastern Nigeria. One hundred and one participants (44 male and 58 female) were recruited into the study. QoL domains were assessed using the stroke-specific Health-Related Quality of Life in Stroke Patients (HRQOLISP). The physical domain was significantly lower than other domains measured (mean = 2.52, SD = 0.76), contributing to poor quality of life. On the other hand, the spiritual domain had the greatest positive influence on QoL (mean = 3.70, SD = 0.50). We found the physical domain was the poorest part of stroke survivors’ stroke experience. The spiritual domain had a positive impact on improving QoL. There is a need for research on interventions relating to the physical rehabilitation of stroke survivors and a review of how the spiritual domain can be enhanced to improve QoL.

## 1. Introduction

Stroke is the second-leading cause of death and the third most common cause of long-term disability worldwide [1]. A stroke, also known as a cerebrovascular accident (CVA), is the medical term for a clinical syndrome rather than a homogeneous condition. There are over 12 million new cases of stroke worldwide, with more than 5 million deaths due to stroke and 6.5 million people becoming permanently disabled [2,3]. The incidence of stroke is expected to increase significantly as the global population of those above 65 years continues to grow by approximately nine million per year [2]. In 2019, there were more than 700 million people in this age group, but this is predicted to increase to over 1.5 billion by 2050; it is expected that two-thirds of them will be living in low-income and middle-income countries (LIMCs) [4]. In Sub-Saharan Africa, Nigeria falls into this category, having the largest country population in Africa with an estimated 218 million people [5].

Furthermore, stroke is a disease of immense public health importance because it has economic and social consequences [1], particularly in LMICs, and is, therefore, a critical health research area [6].

The burden of stroke not only lies in the high mortality figures but also in the increased morbidity, with more than 87% disability in LMICs [3,7]. Over 87% of all deaths from stroke occur in LMICs, with Sub-Saharan Africa (SSA) still maintaining a high stroke incidence [8,9]. Data on SSA show an annual stroke incidence rate of up to 316 cases per 100,000 people, a prevalence rate of 1300 cases per 100,000 people, and a fatality rate of approximately 84% [1]. SSA is estimated to lose more disability-adjusted life years (DALYs) than high-income countries (HICs) [10]. Studies have suggested that in the next few decades, the burden due to stroke in sub-Saharan Africa is likely to increase substantially due to the epidemiological transition in the region from infectious to non-communicable diseases [3,7]. 

This also has substantial financial implications in LMICs such as Nigeria, with a population of over 200 million people and a prevalence rate of 26 strokes per 100,000. The indirect costs of strokes, excluding nursing care, are estimated at an average of $1.1 billion (N173.8 billion) each year in Nigeria [11,12]. With that said, research into stroke survivors’ quality of life (QoL) has received a lot of attention in HICs but only recently in Africa, specifically Nigeria [3,13]. 

Since the burden of stroke is expected to increase significantly in LIMCs, there is a need to better understand the condition [7,14]. This indicates a knowledge gap in the literature, and stroke causes a significant decrease in QoL [10]. This study focuses on Southeastern (SE) Nigeria because most QoL research in Nigeria is focused on the Southwestern region of the country. For example, a systematic review in Nigeria on the impact of stroke on QoL revealed that only one study was conducted in the SE region, with 15 in southwestern Nigeria [15]. SE Nigeria is of particular importance given the history of the Biafran war in the 1960s with the accompanying regionwide starvation, which may contribute to stroke outcomes [16]. Those who were children during the war are now approximately 60 years of age, which may have implications for stroke management in that region. Therefore, the aim of this study is to assess the QoL of stroke survivors in SE communities in Nigeria using the HRQOLISP. 

## 2. Materials and Methods

### 2.1. Study Population and Recruitment

One hundred and one stroke survivor participants were recruited from (a) Amaku Chukwuemeka Odumegwu Ojukwu University Teaching Hospital based in Awka (the main teaching hospital within the community); (b) Nnamdi Azikiwe University Teaching Hospital (NAUTH), Nnewi, Anambra; and (c) Stroke Action Nigeria (SAN), a non-profit organisation dedicated to stroke survivors, founded by Rita Melifonwu, a nurse (CEO), in Onitsha in the year 2000. Participant recruitment for the study took place between December 2019 and January 2021. Convenience sampling was used as a recruitment tool. These participants were survivors who visited the clinics and were approached and invited to participate. To participate, all survivors who met the inclusion criteria (Table 1) were invited and contacted. Individuals that do not meet the inclusion criteria were excluded from the study (Table 2) 

### 2.2. Data Collection

The HRQOLISP is a robust Health Related Quality of Life (HRQOL) questionnaire for patients with stroke and has been validated by the WHO [17]. This was administered to each participant who met the inclusion criteria within the locations specified that were geographically local to the participant. It is a measurement of the health status of individuals and can be used to identify and prioritise areas of need for individual patients and patients with special needs. This outcome measure is also important for identifying the determinants of good and poor prognosis in patients with stroke [18]. 

The HRQOLISP is a 40-item scale outcome measure that encompasses two dimensions and seven domains. The physical dimension includes physical, psychological, cognitive/intellectual, and eco-social or Activities of Daily Living (ADL) domains, while the spiritual dimension comprises soul, spirit, and spiritual interaction domains. The questionnaires were completed with the researcher and research assistant (who signed a Non-Disclosure Agreement) in line with the participants’ choices for each item. This procedure lasted approximately 30–45 min.

### 2.3. Data Analysis

Scores for each domain were summed, and this indicated the severity of the stroke with stratification into severe stroke, moderate stroke, and mild stroke outcomes. The arithmetic mean of the various domain scores was calculated and compared at the subscale level. The seven domains were used as dependent variables in this study, while age, income, gender, stroke type, and physical impairment were used as independent variables. Pearson correlations, independent *t*-tests, one-way analysis of variance (ANOVA), and multiple regression were used to determine the relationships between these variables. The Statistical Package for the Social Sciences (SPSS) version 22 was used to analyse the data. 

### 2.4. Ethical Considerations

This multi-centre cross-sectional study was approved by the University of East London Ethical Research Committee (ERC). Approvals were also gained from the participating institutions in Nigeria. Participants’ data were obtained with permission from the central offices of the registries of the above institutions. Letters of consent were provided for the administrative directors. Volunteers who matched the inclusion criteria were invited. Participants were provided with an information sheet and a consent form. 

## 3. Results

Table 3 below presents a summary of the socio-demographic profile and the type and number of strokes of the survivors who participated in this study. The sample comprised a total of n = 101 participants, with 52.5% males and 47.5% females. The mean age of our sample was 61.01 years (SD = 12.74), and most were married (85%). One-third of the survivors (n = 32, 32.7%) had primary education while 30% had a university degree. More than half of the patients had a monthly income between N10 K and N100 K (equivalent to £10–£100). Haemorrhage accounted for 23.8% of the strokes, while ischaemic accounted for 68.3% of the strokes. More than 80% of our sample had one stroke, while 16.8% suffered two stroke episodes.

**Figure 1:**
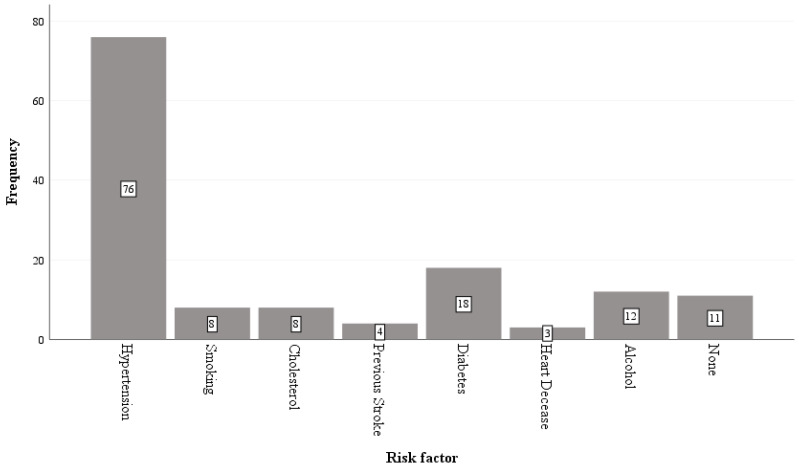
Bar plot of risk factors of stroke indicated by respondents.

Table 4 present frequency of risk factors that contribute to stroke episode among participants. Hypertension having the highest percentage in comparison to known other risk factors. Figure 1 present stroke risk factors using bar plot. 

Table 5 depicts the descriptive statistics of the seven domains (dependent variables): physical, emotional, intellectual, soul, eco-social, spiritual interaction, and spirit. The HRQOLISP scores for each domain ranged from one to five, with the higher scores reflecting greater QoL and the lower scores reflecting poor QoL. The arithmetic mean of the various domains scores was calculated. The descriptive statistics of each dimension indicated a relatively low mean score for the physical dimension of the HRQOLISP (mean = 2.52, SD = 0.76), with the mean values for other dimensions being comparable. Specifically, the spirit and soul domains showed relatively higher mean values (mean = 3.70, SD = 0.70).

The lowest-scoring domain demonstrating poor QoL was the physical domain (mean = 2.52, SD = 0.76), whereas the highest-scoring domain demonstrating better QoL was the spirit domain (mean = 3.70, SD = 0.70). This demonstrates that the physical, emotional, and intellectual domains were the most affected HRQOL domains for stroke survivors and had the utmost impact on their QoL in the SE communities of Nigeria.

Pearson’s correlations were used to assess the significance of the relationships between the HRQOL domains. Table 6 presents the correlation matrix of the HRQOL domains. 

The correlation analysis indicates that the physical dimension did not significantly correlate with any other dimension (*p* ≥ 0.05). However, the emotional (psychological) dimension at (*p* < 0.05) had a significant positive correlation with the intellectual (*p* < 0.01) and soul (*p* < 0.01) dimensions. The intellectual (cognitive) dimension significantly correlated with the soul (*p* < 0.01) dimension and had a significant positive correlation with the eco-social (ADLs) (*p* < 0.01) dimension but a negative correlation with the spirit (*p* < 0.01) dimension of HRQOL. The spiritual interaction was significantly associated with the spirit dimension. The emotional (psychological) dimension had a significant positive correlation with the intellectual (cognitive) and soul dimensions, suggesting that high scores for the emotional (psychological) dimension are associated with high scores for the latter two dimensions. The intellectual (cognitive) dimension had a significant positive correlation with the soul, spirit, and eco-social (ADLs) dimensions, which suggests high intellectual (cognitive) scores are associated with high eco-social (ADLs) scores. However, the intellectual (cognitive) dimension had a negative significant correlation with the spirit domain, which suggests that increased intellectual (cognitive) ability is not associated with spiritual ability. Therefore, high intellectual (cognitive) scores were not associated with the spiritual dimension. 

**Figure 2:**
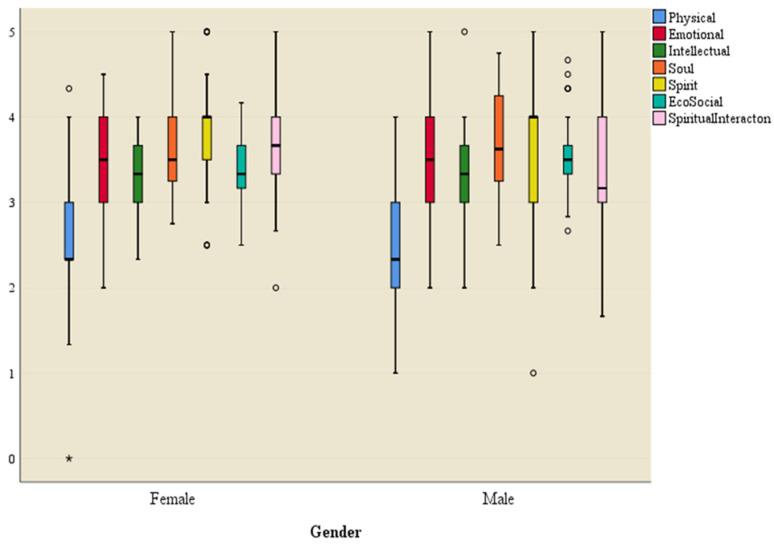
Box plot of HRQOL dimensions for male and female subjects. * = Significant outlier data point outside the 25–75 percentile for female physical domain. o = non-significant outlier data points outside the 25–75 percentile for QoL domains.

An independent samples *t*-test was used to test the significance of the difference in the mean score of each dimension of the HRQOL construct between males and females. Figure 2 shows the box plot of each dimension of the HRQOL construct for males and females.

Table 7 presents a summary of the results of the comparison of each dimension between the male and female groups.

Results of the independent samples *t*-test indicated that there is no significant difference in the mean score of each dimension of the HRQOL construct between male and female groups (*p* ≥ 0.05). Gender did not have a significant effect on any dimension of the HRQOL construct. A single-factor ANOVA model was constructed and tested to assess the effect of income on the dimensions of HRQOL. 

Table 8 presents descriptive statistics of the different dimensions of HRQOL along with a summary of the results of the ANOVA test.

The results of the ANOVA test indicated that there was no significant difference in mean scores across income levels for the physical, soul, spirit, eco-social, and spiritual interaction dimensions of HRQOL. This reveals that there is no significant effect of income on the dependent variables—the HRQOL dimensions mentioned above. However, the emotional and intellectual dimensions of HRQOL revealed a significant difference across income levels for the emotional (F (3, 87) = 3.171, *p* = 0.028) and intellectual dimensions (F (3, 87) = 4.124, *p* = 0.009).

**Figure 3:**
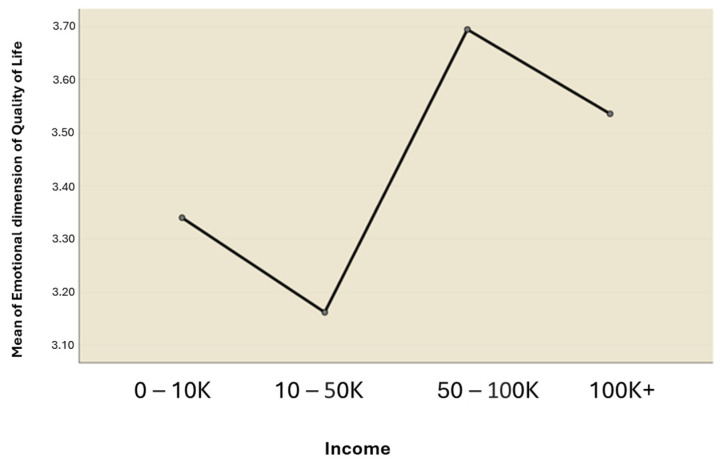
Mean plot of emotional dimension of quality of life across income levels.

**Figure 4:**
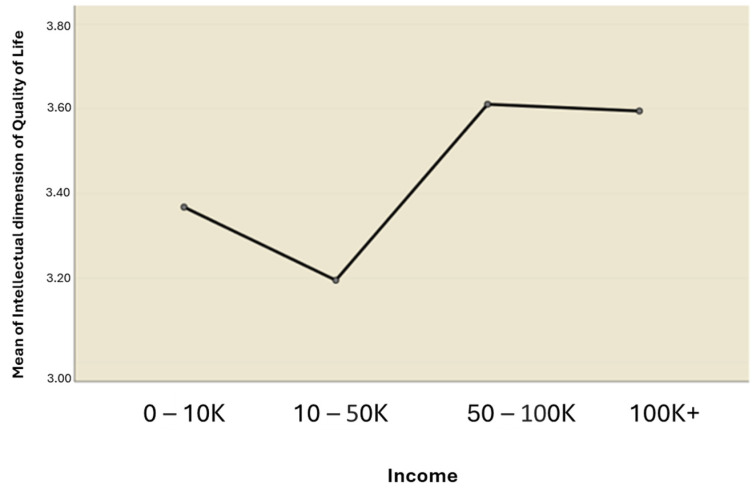
Mean plot of Intellectual dimension of quality of life across income levels.

Scheffe’s post hoc test was used to make possible contrasts between group means and for unplanned comparisons. The results of the test indicated significant differences in the emotional and intellectual dimensions of HRQOL between the N10 K–50 K* and N50 K–100 K* income levels (Figure 3 and Figure 4). Specifically, regarding the emotional dimension (Figure 3), the mean score for subjects at the N10 K–50 K* income level (M = 3.16, SD = 0.57) was significantly lower (mean difference = −0.532, 95% CI: −1.056 to −0.009) than that of subjects at the N50 K–100 K* income level (M = 3.69, SD = 0.75). Furthermore, concerning the intellectual dimension (Figure 4), the mean score for those at the N10 K–50 K* income level (M = 3.20, SD = 0.45) was significantly lower (mean difference = −0.416, 95% CI: −0.810 to −0.022) than that of the subjects earning N50 K–100 K* (M = 3.61, SD = 0.60). N* = Naira (Nigerian currency).

An independent samples *t*-test was used to test the significance of the difference in the mean scores of each dimension of the HRQOL construct between the sides affected by the stroke (stroke orientation). 

**Figure 5:**
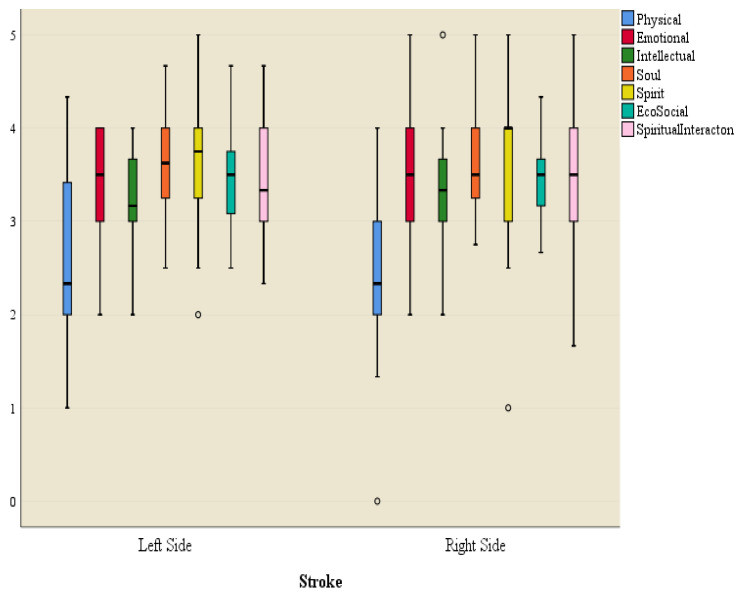
Box plot of dimensions of quality of life for left and right-side hemiparesis. o = non-significant outlier data points outside the 25–75 percentile for QoL domains based on sides of stroke impact on participants.

Figure 5 is a box plot of each dimension of the HRQOL construct for the two sides (left and right hemiparesis). 

Table 9 presents a summary of the results of the comparison of each dimension between the two groups. 

Results of the independent samples *t*-test indicated that there is no significant difference in the mean score of each dimension of the HRQOL construct between left and right hemiparesis affected by the stroke. The side of the body affected by the stroke did not show a significant effect on any dimensions of the HRQOL construct. 

An independent samples *t*-test was used to test the significance of the difference in the mean score of each dimension of the HRQOL construct between the haemorrhagic and ischaemic stroke types. 

**Figure 6:**
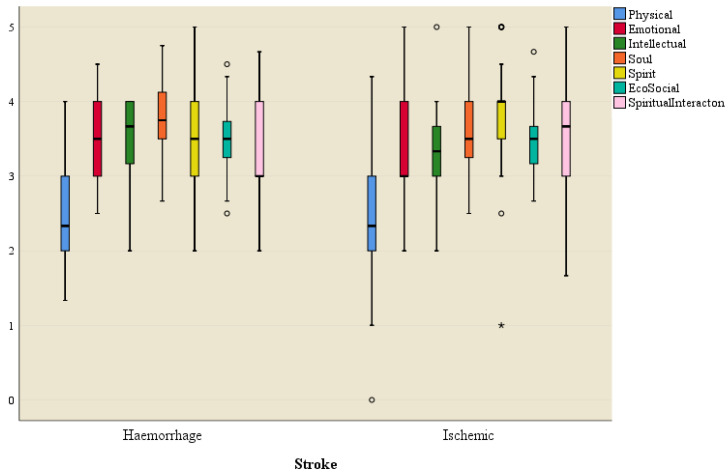
Box plot of dimensions of quality of life for types of strokes. * = Significant outlier data point outside the 25–75 percentile for QoL domain based on type of stroke. o = non-significant outlier data points outside the 25–75 percentile for QoL domains based on type of stroke.

Figure 6 shows a box plot of each dimension of the HRQOL construct for the two types of strokes. 

The results of the independent samples *t*-test indicated no significant difference in the mean scores of each HRQOL dimension construct between haemorrhagic and ischaemic types of strokes (Table 10). The type of stroke did not have a significant effect on any dimension of the QOL construct. 

A linear regression model was constructed and tested to evaluate the significance of the predictive effect of age, gender, income level, type of stroke, and body side affected due to stroke. Specifically, a general linear model (GLM) was applied, taking gender, income, type of stroke, and body side as categorical predictors and age as a continuous predictor.

Table 11 presents the results of the GLM model (with model effects of each predictor, along with the test for its significance). The results of the GLM analysis revealed significant models and predictors of the spiritual interaction dimension of HRQOL. The R-squared value was 0.179, indicating that 17.9% of the variance in the spiritual interaction dimension can be explained by the predictors. Among the predictors, gender and income were statistically significant but with small effects (η^2^ < 0.20) on the spiritual interaction dimension for gender (F (1, 75) = 4.341, *p* = 0.041) and income (F (3, 75) = 4.067, *p* = 0.010). Specifically, adjusting for the effect of other predictors, the mean spiritual interaction level was significantly higher for females compared with males (mean difference = 0.277, t (75) = 2.083, *p* = 0.041, 95% CI for mean difference: 0.012–0.542).

For income levels, Bonferroni adjusted comparisons indicated that the subjects in the high-income group of 100 K or more had significantly higher mean spiritual interaction scores compared with subjects in the 10 K–50 K income group (mean difference = 0.701, 95% CI for mean difference: 0.156–1.247).

E = standard error, η^2^ is the measure of effect size associated with the model parameter and K* = Income in Naira (Nigerian currency). The GLM analysis indicated that the models for the physical, emotional, intellectual, soul, spirit, and eco-social dimensions of QoL were statistically not significant. This implies that none of the predictor variables had a statistically significant effect on these dimensions of the HRQOL construct. 

The results of the GLM analysis revealed a significant effect of gender and income on the spiritual interaction dimension of the QoL of subjects. Age, gender, income, type of stroke, and body side affected by the stroke failed to show a significant predictive effect on the physical, emotional, intellectual, soul, spirit, and eco-social dimensions of QoL. 

The variables and relationships that predict the impact of stroke on the QoL of stroke survivors in Nigeria’s SE communities are determined in the following results. Stroke had a twofold effect on QoL in this study: a good or poor influence. The descriptive statistics for HRQOL revealed that the lowest-scoring domain that demonstrated a negative impact on QoL was the physical domain (mean = 2.52, SD = 0.76), whereas the highest-scoring domain that demonstrated a positive impact on QoL was the spirit domain (mean = 3.70, SD = 0.70). This shows that the physical, followed by the emotional (psychological) and intellectual (cognitive) domains are the most affected of the HRQOL domains of the stroke survivors in this sample. This ultimately had the most impact on the QoL of the stroke survivors in the SE communities of Nigeria. 

Pearson’s correlation was used to analyse the relationships between the variables, revealing that the emotional (psychological) domain (*p* < 0.05) is associated with the intellectual (cognitive) domain (*p* < 0.01). This means that high scores for the former are associated with high scores for the latter. The intellectual (cognitive) domain is also associated with the eco-social (ADLs) (*p* < 0.01) and soul (*p* < 0.01) domains, which suggests that high scores of the intellectual (cognitive) domain are associated with high scores for the latter two. 

The intellectual (cognitive) domain was negatively correlated with the spiritual domain. In other words, when stroke survivors improved their intelligence (cognitive capacity), they appeared to lose their spiritual orientation.

A significant difference was observed between the emotional (psychological) and intellectual (cognitive) domains for both the 10 K–50 K and 50 K–100 K income levels. Regarding the intellectual (cognitive) domain, the mean score of the subjects at the 10 K–50 K income level (M = 3.20 SD = 0.45) was significantly lower than the mean score for those earning 50 K–100 K (M = 3.61 SD = 0.60), with a mean difference of −0.416 (95% CI = −0.0810 to −0.022).

The general linear regression test analysed the associated predictors and revealed that the predictors of gender (F (1, 75) = 4.341 *p* = 0.041)) and income (F (3, 75) = 4.067, *p* = 0.010) had a statistically significant effect (n^2^ < 0.20) on the spiritual interaction domain (F (1, 75) = 4.341 *p* = 0.041).

The results of the GLM analysis revealed predictors of the spiritual interaction domain of the HRQOL—with the R-squared value being 0.179, indicating 17.9% of the variance in the spiritual interaction domains. This suggests that the GLM analysis revealed that income and gender were the determinants of HRQOL in this study, most particularly in the spiritual interaction domain.

## 4. Discussion

The aim of this study was to assess the QoL of stroke survivors in SE communities in Nigeria using the HRQOLISP questionnaire. The age of the participants in this study showed similarity to studies conducted in low-income countries, which frequently involve those between middle and early older age [19,20,21]. Although previous studies have found that stroke affects more men than women [22,23], this study found that gender does not have a significant impact on the QoL of stroke survivors and found that the impact of stroke is similar in both genders. 

The results of this study build on existing evidence. However, a study reports a mean age of 63.4 years for stroke survivors, which is higher than in our study by 1.9 (age group: 74–84 years) versus 1.00 (age group: 55–63 years) per 1000 population [24]. This may be due to reports of low average age of a stroke attributed to a variety of factors, including decreased life expectancy and a high prevalence of fatal stroke in LMICs [7,25]. In contrast, the mean age and peak prevalence of stroke in high-income countries have been reported to be higher than those in LMICs [1,25]. In the German population, the mean age for stroke survivors was higher—69 years. This disparity may be due to the relatively long-life expectancy in high-income communities [25]. The male prevalence of stroke noted in our study is comparable to data obtained in SSA studies [7,25]. 

Africa appears to have the highest incidence, prevalence, and case fatality rates of stroke [1]. The age-specific stroke incidence is relatively higher in younger age groups in sub-Saharan Africa, as noted in the study. It is further argued that people of African descent experience strokes at a younger age and have worse outcomes [26,27]. 

Hypertension was the most significant stroke risk factor in our study, more than three-fourths of our sample (n = 76, 76.8%) had hypertension, followed by diabetes (n = 18, 28.2%). Studies in SSA have also shown that hypertension is a major risk factor for stroke. For example, in Mozambique, 86.6–96.0% of survivors of stroke had hypertension before hospital admission [28]. Hypertension is the strongest risk factor after age, and individuals suffering from it are three or four times more likely to have a stroke [7,29].

This study also observed other potential stroke risk factors, such as smoking (8.1%), cholesterol (8.1%), heart disease (3.0%), and alcohol (12.1%). However, in comparison with studies in the UK and Germany [1,30], the prevalence of cardiovascular disease, high cholesterol, and ischaemic diseases amongst survivors with stroke is approximately (20–30%), much higher than what was observed in the participants with stroke in the SE communities of Nigeria. This disparity indicates that the geographical distribution of subtypes of stroke has an important effect on stroke [1,30]. Due to the limited data, we could not investigate the relationship between stroke risk factors and stroke subtypes. Other studies have reported that haemorrhagic stroke is more associated with hypertension, whereas ischaemic stroke is more affiliated with smoking and cardiac diseases [1,25,31]. However, our study found 68% of ischaemic type of stroke and only 24% haemorrhagic type of stroke. Our findings align with a Ghanaian study [6] in which ischaemic strokes accounted for 78% of all cases. 

Although hypertension is more commonly associated with haemorrhagic stroke, it also causes an ischaemic stroke. Nevertheless, additional research is required to understand the aforementioned. The higher prevalence of ischaemic stroke in the SE communities may also be due to the adoption of the Western lifestyle. The negative health impacts of Western lifestyles on the health of rural communities to urban/semi-urban cities over the years have been documented [32,33].

Studies have shown that the QoL of stroke survivors is consistently influenced by stroke severity [7,25,31]. However, the distribution of stroke types in our study indicated the impact of haemorrhagic stroke (23.8%), while ischaemic stroke (69%) showed no significant effect on the dimensions of QoL. In contrast, haemorrhagic stroke survivors had considerably higher QoL during poststroke periods [23]. However, Owolabi et al. [17] and Owolabi et al. [34] found no significant correlation between stroke type and QoL in their respective studies. Our findings correspond with these later findings. 

The lowest scoring domain (negative) QoL was the physical domain (mean = 2.52, SD = 0.76). The highest scoring domain (positive) QoL was the spirit domain (mean = 3.70, SD = 0.70. This demonstrates that the physical, emotional, and intellectual domains were the most affected HRQOL domains and had the most impact on the QoL of stroke survivors in the SE communities of Nigeria. This implies the need to improve physical therapy/rehabilitation approaches using personalised and multimodal strategies tailored to the specific needs of each patient. Although the highest scores were discovered in the spiritual domains, this suggests that drawing on one’s faith or religion throughout the time of recovery is essential since doing so can aid healing. Previous studies have demonstrated variability in scores regarding domains. Nevertheless, in contrast to our study, a study with samples from Denmark and Norway reported more problems in the cognitive, social, and emotional domains than in the physical one [35]. This suggests that the participants are from an HIC with improved rehabilitation facilities and infrastructure, which may be one cause. Another study found personality and thinking (intellectual) to be among the domains with the highest scores [36]. While some Nigerian studies [13,37] have revealed that ‘social, family and physical functioning’ are areas of QoL most affected. A study reveals the area most severely affected by the QoL is ‘emotions’ [38]. In contrast, the findings of a Swedish study indicated that concerns about functioning were more widespread in the physical domains than they were in the cognitive and social domains [39]. However, one potential reason for these discrepancies may be related to the contrasts and similarities that exist due to these countries being HICs in comparison to LMICs. Due to a lack of access to rehabilitation services, it is now clearly obvious that survivors of stroke in these LMICs receive very modest therapeutic treatments to aid with recovery. This is only one possible explanation. However, discrepancies in HRQOL ratings may also be attributed to the range of patient groups evaluated, in addition to the diverse recruitment techniques employed by the various stroke hospitals and research organisations [39].

HRQOL associations may vary over time after stroke; this may depend on whether different aspects or components of the multidimensional HRQOL are being considered. The correlation analysis indicates that the physical dimension did not significantly correlate with any other dimension (*p* ≥ 0.05). This may be due to the small sample size used for the study. However, this agrees with other studies that have identified a reduced correlation with other dimensions [31,39].

This study found that the emotional (psychological) domain had a significant positive correlation with the intellectual (cognitive) domain. Our findings suggest that the physical, emotional, and intellectual domains were most negatively impacted by stroke. This, therefore, suggests that emotions and intellect are related. Consequently, these domains had the biggest impact on the overall QoL of the survivors in the research study. This is in accordance with studies such as by Pinkney and colleagues [40], who reported that the cognitive domain is one of the two domains most significantly affected by stroke. However, Pinkney et al. [40] suggest that 30% of stroke victims have computer tomography (CT) findings showing atherosclerotic changes in small vessels or have white matter disease. These white matter changes are often implicated in intellectual (cognitive) decline [40]. It would have been interesting to know the outcomes of the CT scans performed on the stroke survivors who participated in our research study. To be more specific, it would have been intriguing to determine whether the lack of white matter alterations in these individuals explains their poor HRQOL. This work may be beneficial for future research targeting people who have already had CT scans or who have the financial means to do so, as most survivors in SE Nigeria cannot afford a CT scan. Although more studies have been conducted in the Southwestern part of Nigeria, general findings are similar to those of our study, with stroke survivors presenting a low QoL [41]. This may be an indication of Nigeria’s struggling healthcare system in all geopolitical zones of the country.

There was a significant association found between the spiritual interaction domain and QoL when analysing the HRQOL component that pertains to one’s spirituality. In Pinkney et al.’s study [40], stroke survivors had significantly reduced HRQOL scores across the board, apart from the category of spiritual engagement. 

The effect of age, gender, income, afflicted body side (stroke orientation), and stroke type on the mean score of each dimension of the HRQOL construct, except for gender, showed no significant differences. The results build on existing evidence from studies such as those by Owolabi et al. [31] and Sampane-Donkor et al. [6], who suggest that gender has no significant effect on HRQOL. However, female survivors with stroke had better HRQOL in the spirit and spiritual interaction domains than their male counterparts in the above studies. Other similar studies have equally shown that gender has no influence on QoL [36]. Contrarily, Enato et al. [42] found that Nigerian women had a significantly poorer QoL when compared to men based on activities of daily living. Further studies are required using comparable instruments and methodologies to clarify the effect of gender on different HRQOL domains [6,30].

### Strength and Limitation

This study was useful for further understanding the impact of stroke on quality of life from the perspectives of stroke survivors in the Southeast communities of Nigeria. Thus, there can be confidence that the findings reflect issues that are important to this population. This is particularly important given that the SE region of Nigeria has not been as explored as the South Western part of Nigeria. 

A limitation of the study is that the cross-sectional design does not enable the assessment of changes in HRQOL. Due to the cross-sectional nature of this study, it was impossible to capture changes in participants’ characteristics over time. Despite this, the study proved valuable in assessing the impact of stroke on survivors’ quality of life in Nigeria’s SE region.

The questionnaire provided seven domains’ scores rather than an amalgamated score. Such a score would need weighting of the domains, and that would be done as a future extension of this research, which would entail a larger scale. However, there were limited descriptive data on the characteristics of the quantitative sample, although this was done to minimise the data collection time. Additionally, when using lengthy instruments, one must consider the burden on patients and staff, as well as the viability in terms of the resources available. The participants were mostly older people with a variety of comorbid illnesses. Given this population’s fragility and advanced age, HRQOL instruments should be assessed for their clarity and capacity to distinguish between the impacts of illness and ageing [43]. In addition to these, we acknowledge that our sample size may have an impact on generalizing the findings from this study. Other limitations that may impact our findings include the use of convenience sampling and the distribution of the questionnaire in the English language. 

To further improve support for stroke survivors in Nigeria, it is recommended to emphasise the importance of rehabilitation practitioners supporting stroke survivors to engage in meaningful self-selected social activities, given the importance of the physical domain as found in this study. Given the multi-professional support required to improve rehabilitation plans for stroke survivors, it is recommended that physical and emotional support services are provided to stroke survivors. This can be achieved through a collaborative effort between the occupational therapy department and mental health support teams within healthcare in Nigeria. However, the availability of these services is limited in the Nigerian healthcare system, especially the mental health professions. However, this will be a key way to support stroke survivors with the physical and emotional aspects of their QoL. Furthermore, survivors can be supported to make a greater contribution to their treatment; the importance of stroke survivors having the freedom and autonomy to set their own goals within rehabilitation has been reported [44]. Further research is needed to assess the change in QoL over a long period of time using a longitudinal study design. This can provide valuable insight into changes in QoL as the years living after the stroke event progress; this will help with designing interventions according to the health needs of survivors.

## 5. Conclusions

In summary, stroke has a multifaceted effect on HRQOL and is more pronounced in the physical and spiritual domains. This study revealed that none of the independent variables had significant relationships or correlations with the dependent variables. This may be due to the cross-sectional design of the study, which necessitated a larger sample size. Indeed, additional research on the linkages and correlations between these variables is required.

## Figures and Tables

**Table 1:** Inclusion criteria.

•	Patients with Definite Clinical and Radiological Stroke Diagnosis.
•	Patients involved in a stroke rehabilitation programme.
•	Patients who had a stroke within one or two years prior to the time of contact with the investigator.
•	Patients who can communicate verbally and in the English language.
•	Patients who are independently mobile with or without an aid, such as a scooter, wheelchair (powered or nonpowered), Zimmer frame, walking stick, or cane.

**Table 2:** Exclusion criteria.

•	Patients Who Do Not Fulfil the Inclusion Criteria.
•	Patients with other medical conditions that are neither risk factors for nor complications of stroke but could interfere with HRQOL.
•	Patients who are acutely ill and cannot withstand the rigour of a robust questionnaire or interview.
•	Patients with ambiguous stroke diagnosis.
•	Patients who are unable to communicate effectively for interview purposes.

**Table 3:** Demographic profile and type of stroke suffered by participants.

Variable		n	%	Mean	SD
Age		101	100	61.01	12.74
Gender	Male	53	52.5		
	Female	48	47.5		
Education	Primary	32	31.7		
	Secondary	20	19.8		
	College	12	11.9		
	University	30	29.7		
	Others	7	5.0		
Marital status	Single	5	5.0		
	Married	85	85.0		
	Divorced	1	1.0		
	Widowed	8	7.9		
	No response	2	2.0		
Income *	0–10 K	25	24.8		
	10 K–50 K	35	34.7		
	50 K–100 K	18	17.8		
	100 K+	14	13.9		
	No response	9	8.9		
# Current Spouses	0	9	8.9		
	1	90	89.1		
	2	2	2.0		
Type of stroke	Haemorrhage	24	23.8		
	Ischaemic	69	68.3		
	N/S	1	1.0		
	Unknown/Missing	7	7.0		
% Strokes	1	81	80.2		
	2	17	16.8		
	3	3	2.9		

* Income in Nigerian currency, naira (£1 = approx. 1852 naira). # Two male participants are married to more than one spouse at the same time. % This represents number of stroke episodes experienced by participants.

**Table 4:** Risk factors associated with stroke.

Risk Factors *	N	Percentage
Hypertension	76	76.8%
Smoking	8	8.1%
Cholesterol	8	8.1%
Previous Stroke	4	4.0%
Diabetes	18	18.2%
Heart–Disease	3	3.0%
Alcohol	12	12.1%
None	11	11.1%

* Multiple responses. Distribution of stroke risk factors amongst Southeastern Nigerian stroke survivors.

**Table 5:** Descriptive statistics of quality-of-life dimensions.

	N	Minimum Score	Maximum Score	Mean	SD
Physical	101	2.22	4.33	2.52	0.76
Emotional	101	2.00	5.00	3.40	0.66
Intellectual	101	2.00	5.00	3.39	0.53
Soul	101	2.50	5.00	3.68	0.56
Spirit	101	1.00	5.00	3.70	0.70
Eco Social	101	2.50	4.67	3.47	0.40
Spiritual Interaction	101	1.67	5.00	3.50	0.64

**Table 6:** Correlation matrix of Health-Related Quality-of-Life dimensions.

	Physical	Emotional	Intellectual	Soul	Spirit	Eco-Social	Spiritual Interaction
Physical							
Emotional	0.051						
Intellectual	0.111	0.647 **					
Soul	−0.140	0.598 **	0.458 **				
Spirit	0.043	−0.181	−0.280 **	−0.210 *			
Eco-social	−0.058	0.247 *	0.273 **	0.249 *	−0.140		
Spiritual Interaction	0.170	−0.122	−0.078	−0.174	0.360 **		

Note: * significant at 0.05 level, ** significant at 0.01 level.

**Table 7:** Effect of gender on dimensions of health quality of life.

	Gender	N	Mean	SD	t	df	*p*	95% CI for Mean Difference	
Physical	Male	52	2.44	0.70	−1.17	98	0.24	(−0.48	0.12)
	Female	48	2.62	0.83					
Emotional	Male	53	3.39	0.66	−0.13	98	0.90	(−0.28	0.24)
	Female	47	3.40	0.66					
Intellectual	Male	53	3.41	0.54	0.31	98	0.76	(−0.18	0.25)
	Female	47	3.38	0.53					
Soul	Male	53	3.72	0.61	0.65	99	0.52	(−0.15	0.29)
	Female	48	3.64	0.51					
Spirit	Male	52	3.67	0.76	−0.32	98	0.75	(−0.32	0.23)
	Female	48	3.72	0.64					
Eco Social	Male	52	3.54	0.41	1.78	98	0.08	(−0.02	0.30)
	Female	48	3.40	0.38					
Spiritual Interaction	Male	53	3.43	0.73	−1.26	98	0.21	(−0.42	0.09)
	Female	47	3.59	0.53					

**Table 8:** Effect of income on dimensions of quality of life.

Dimension	Income	N	Mean	SD	F	*p*
Physical	0–10 K	25	2.68	0.93	0.846	0.472
	10 K–50 K	34	2.36	0.60		
	50 K–100 K	18	2.46	0.79		
	100 K+	14	2.51	0.74		
Emotional	0–10 K	25	3.34	0.61	3.171	0.028
	10 K–50 K	34	3.16	0.57		
	50 K–100 K	18	3.69	0.75		
	100 K+	14	3.54	0.63		
Intellectual	0–10 K	24	3.37	0.41	4.124	0.009
	10 K–50 K	35	3.20	0.45		
	50 K–100 K	18	3.61	0.60		
	100 K+	14	3.60	0.49		
Soul	0–10 K	25	3.57	0.51	0.621	0.603
	10 K–50 K	35	3.65	0.63		
	50 K–100 K	18	3.70	0.56		
	100 K+	14	3.82	0.42		
Spirit	0–10 K	25	3.70	0.82	0.266	0.849
	10 K–50 K	35	3.69	0.71		
	50 K–100 K	18	3.69	0.55		
	100 K+	13	3.88	0.77		
Eco Social	0–10 K	24	3.32	0.42	1.641	0.186
	10 K–50 K	35	3.52	0.34		
	50 K–100 K	18	3.56	0.46		
	100 K+	14	3.44	0.40		
Spiritual Interaction	0–10 K	25	3.53	0.55	0.748	0.527
	10 K–50 K	35	3.43	0.59		
	50 K–100 K	17	3.55	0.72		
	100 K+	14	3.71	0.63		
	Total	91	3.52	0.61		

**Table 9:** Effect of stroke orientation (affected body side) on dimensions of quality of life.

	Part	N	Mean	SD	t	df	*p*	95% CI for the Mean Difference	
Physical	Left	34	2.64	0.85	1.30	92	0.20	(−0.11	0.54)
	Right	60	2.43	0.72					
Emotional	Left	33	3.33	0.54	−0.67	92	0.51	(−0.37	0.18)
	Right	61	3.43	0.69					
Intellectual	Left	34	3.28	0.54	−1.53	92	0.13	(−0.37	0.05)
	Right	60	3.44	0.47					
Soul	Left	34	3.64	0.58	−0.73	93	0.47	(−0.33	0.15)
	Right	61	3.73	0.55					
Spirit	Left	34	3.71	0.66	0.15	92	0.88	(−0.28	0.33)
	Right	60	3.68	0.74					
Eco Social	Left	33	3.48	0.53	0.14	92	0.89	(−0.16	0.19)
	Right	61	3.47	0.32					
Spiritual Interaction	Left	34	3.50	0.58	0.06	93	0.95	(−0.26	0.28)
	Right	61	3.49	0.66					

**Table 10:** Effect of type of stroke on dimensions of quality of life.

	Stroke	N	Mean	SD	t	df	*p*	95% CI for Mean Difference	
Physical	Haemorrhage	24	2.45	0.86	−0.14	90	0.89	−0.38	0.33
	Ischemic	68	2.48	0.70					
Emotional	Haemorrhage	24	3.54	0.49	1.33	90	0.19	−0.10	0.53
	Ischemic	68	3.33	0.72					
Intellectual	Haemorrhage	24	3.50	0.50	1.27	90	0.21	−0.09	0.42
	Ischemic	68	3.34	0.55					
Soul	Haemorrhage	24	3.80	0.51	1.15	91	0.25	−0.11	0.42
	Ischemic	69	3.65	0.58					
Spirit	Haemorrhage	23	3.52	0.70	−1.55	90	0.12	−0.60	0.07
	Ischemic	69	3.78	0.70					
Eco Social	Haemorrhage	24	3.48	0.48	0.03	90	0.98	−0.18	0.19
	Ischemic	68	3.47	0.36					
Spiritual Interaction	Haemorrhage	24	3.31	0.64	−1.69	90	0.09	−0.55	0.04
	Ischemic	68	3.56	0.63					

**Table 11:** Predictive effect of age, gender, and stroke side on dimensions of quality of life.

Dimension of QOL	Predictor	β (SE)	*p*	95% CI of β	η^2^	R^2^	Adj. R^2^
Physical	Gender (female)	0.08 (0.18)	0.66	(−0.28, 0.45)	0.003	0.095	0.009
	Age	−0.01 (0.01)	0.09	(−0.03, −0.002)	0.039		
	Income						
	0–10 K	0.07 (0.28)	0.79	(−0.48, 0.63)	0.001		
	10 K–50 K	−0.28 (0.28)	0.31	(−0.83, 0.27)	0.014		
	50 K–100 K	−0.14 (0.30)	0.64	(−0.74, 0.46)	0.003		
	Body impaired (left)	0.20 (0.18)	0.27	(−0.16, 0.59)	0.02		
	Type of stroke (Haemorrhage)	−0.26 (0.20)	0.21	(−0.65, 0.15)			
Emotional	Gender (female)	0.03 (0.16)	0.83	(−0.28, 0.35)	0.001	0.135	0.053
	Age	−0.003 (0.01)	0.61	(−0.02, 0.009)	0.003		
	Income						
	0–10 K	−0.27 (0.24)	0.26	(−0.74, 0.20)	0.017		
	10 K–50 K	−0.36 (0.23)	0.13	(−0.83, 0.11)	0.031		
	50 K–100 K	0.17 (0.26)	0.52	(−0.35, 0.68)	0.006		
	Body impaired (left)	−0.13 (0.15)	0.42	(−0.43, 0.18)	0.009		
	Type of stroke (Haemorrhage)	0.21 (0.17)	0.22	(−0.13, 0.56)	0.02		
Intellectual	Gender (female)	−0.02 (0.11)	0.86	(−0.25, 0.21)	0.001	0.151	0.071
	Age	−0.001 (0.004)	0.82	(−0.01, 0.01)	0.001		
	Income						
	0–10 K	−0.23 (0.17)	0.19	(−0.57, 0.12)	0.023		
	10 K–50 K	−0.32 (0.17)	0.06	(−0.66, 0.02)	0.046		
	50 K–100 K	−0.05 (0.19)	0.79	(−0.42, 0.32)	0.001		
	Body impaired (left)	−0.19 (0.11)	0.09	(−0.41, 0.03)	0.037		
	Type of stroke (Haemorrhage)	0.18 (0.13)	0.17	(−0.08, 0.43)	0.026		
Soul	Gender (female)	0.01 (0.14)	0.92	(−0.26, 0.29)	0.001	0.053	0.035
	Age	−0.005 (0.005)	0.36	(−0.02, 0.006)	0.011		
	Income						
	0–10 K	−0.26 (0.21)	0.23	(−0.68, 0.17)	0.019		
	10 K–50 K	−0.13 (0.23)	0.63	(−0.55, 0.29)	0.005		
	50 K–100 K	−0.11 (0.23)	0.63	(−0.57, 0.35)	0.003		
	Body impaired (left)	−0.12 (0.14)	0.40	(−0.85, 0.40)	0.010		
	Type of stroke (Haemorrhage)	0.14 (0.15)	0.37	(−0.17, 0.45)	0.011		
Spirit	Gender (female)	0.16 (0.18)	0.38	(−0.20, 0.51)	0.011	0.060	0.029
	Age	0.002 (0.007)	0.79	(−0.01, 0.02)	0.001		
	Income						
	0–10 K	−0.28 (0.28)	0.32	(−0.84, 0.27)	0.014		
	10 K–50 K	−0.37 (0.27)	0.18	(−0.92, 0.18)	0.024		
	50 K–100 K	−0.23 (0.30)	0.46	(−0.83, 0.38)	0.008		
	Body impaired (left)	0.19 (0.18)	0.28	(−0.16, 0.54)	0.016		
	Type of stroke (Haemorrhage)	−0.33 (0.20)	0.11	(−0.74, 0.07)	0.035		
Eco-Social	Gender (female)	−0.17 (0.09)	0.08	(−0.37, 0.02)	0.042	0.093	0.007
	Age	0.0001 (0.004)	0.92	(−0.008, 0.007)	0.0001		
	Income						
	N0–10 K*	0.055 (0.15)	0.72	(−0.24, 0.35)	0.002		
	10 K–50 K	0.25 (0.15)	0.09	(−0.04, 0.54)	0.039		
	50 K–100 K	0.23 (0.16)	0.16	(−0.09, 0.55)	0.027		
	Body impaired (left)	−0.06 (0.11)	0.51	(−0.26, 0.13)	0.006		
	Type of stroke (Haemorrhage)	0.10 (0.11)	0.35	(−0.11, 0.31)	0.012		
Spiritual Interaction	Gender (female)	0.28 (0.13)	0.04	(0.01, 0.54)	0.055	0.179	0.102
	Age	0.01 (0.01)	0.16	(−0.003, 0.017)	0.026		
	Income						
	0–10 K	−0.48 (0.20)	0.021	(−0.89, −0.07)	0.069		
	10 K–50 K	−0.70 (0.20)	0.001	(−1.10, −0.30)	0.139		
	50 K–100 K	−0.49 (0.22)	0.029	(−0.99, −0.05)	0.062		
	Body impaired (left)	0.15 (0.13)	0.270	(−0.12, 0.41)	0.016		
	Type of stroke (Haemorrhage)	−0.29 (0.15)	0.053	(−0.58. 0.004)	0.049		

## Data Availability

The data presented in this study are available on request from the corresponding author. The data are not publicly available due to ethical and privacy reasons.
